# GeoFusion-3D: Multi-Scale Geomorphic Feature Fusion for Landslide Scar Detection Using UAV-Mounted LiDAR

**DOI:** 10.3390/s26113557

**Published:** 2026-06-03

**Authors:** Abhudaya Shrivastava, Shelly Gupta, Zoran Obradovic

**Affiliations:** Department of Computer and Information Sciences, The College of Science and Technology, Temple University, Philadelphia, PA 19122, USA; abhudaya.shrivastava@temple.edu (A.S.); shelly.gupta@temple.edu (S.G.)

**Keywords:** UAV LiDAR, zero-shot, 3D point cloud analytics, landslide-candidate patch identification, post-event landslide susceptibility, geomorphic anomaly detection, depth anomaly modeling, unsupervised clustering, rapid terrain instability mapping, geospatial machine learning

## Abstract

Landslide detection has largely relied on supervised learning or DEM-based representations, which can limit rapid deployment and generalization across heterogeneous terrain. In this work, we present a zero-shot, fully unsupervised framework that identifies landslide-like geomorphic instability candidates from raw UAV-mounted LiDAR, removing the need for labeled data, pre-event baselines, or rasterized terrain abstractions. Our approach is motivated by the observation that landslides manifest as localized geometric inconsistencies in the terrain surface. We capture this through a multi-scale formulation that combines point-level and cluster-level indicators of instability. At the point level, a PCA-based residual depth metric reduces slope-induced bias and highlights surface discontinuities, while local concavity captures terrain depletion patterns. At the cluster level, geomorphometric descriptors such as curvature concentration, surface roughness, elevation discontinuity, and slope variation are extracted using density-aware 3D clustering and integrated through adaptive feature fusion. The resulting probabilistic instability field enables spatially coherent delineation of landslide scars, including rupture boundaries, displaced material, and emerging failure regions. In addition, the detected patches provide useful priors for post-event susceptibility analysis without requiring temporal observations. Experiments across diverse geomorphic settings show that the proposed method improves detection of subtle terrain disturbances compared to DEM-based pipelines and supervised learning approaches, while remaining robust to noise and terrain variability. Overall, this work demonstrates that geometry-driven, unsupervised inference on raw 3D data can serve as a practical and scalable alternative for near real-time landslide detection using UAV-based systems.

## 1. Introduction

Landslides are among the most destructive natural hazards, frequently triggered by rainfall, seismic activity, earthquake shaking, and anthropogenic disturbance, and they cause substantial damage to infrastructure, transportation corridors, and human settlements. Accurate landslide mapping and susceptibility assessment are therefore central to early warning, post-event response, and long-term hazard planning [1,2,3]. However, conventional landslide inventories and susceptibility products often depend on manual interpretation, historical records, or spatially aggregated terrain factors, which can be labor-intensive, region-specific, and difficult to update rapidly after a triggering event [3,4,5].

In recent years, Unmanned Aerial Vehicles (UAVs) equipped with LiDAR sensors have emerged as a powerful platform for acquiring high-resolution three-dimensional (3D) topographic data, including in vegetated, occluded, or topographically complex environments [6,7]. UAV–LiDAR enables dense point-cloud reconstruction and bare-earth terrain representation, offering clear advantages over optical-only sensing when illumination, vegetation cover, or surface texture limits image-based analysis [6,7].

**Figure 1:**
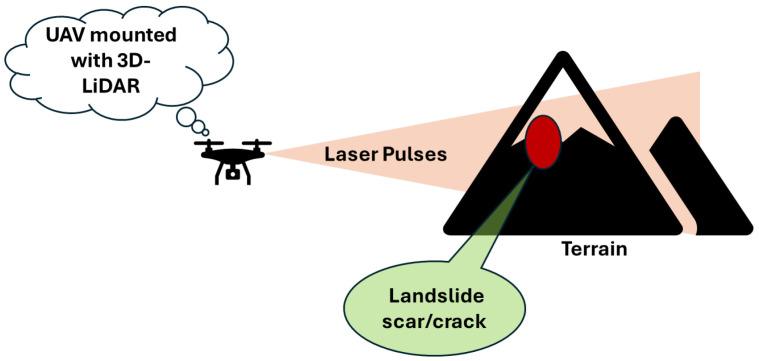
Conceptual illustration of UAV-based 3D LiDAR mapping of landslide-candidate terrain. A UAV emits laser pulses to reconstruct the terrain as a dense 3D point cloud, where landslide-related regions may appear as localized geometric discontinuities, including cracks, scarps, rupture boundaries, displaced surfaces, and debris-like terrain.

As illustrated in Figure 1, a UAV-mounted LiDAR system emits laser pulses toward the terrain and reconstructs a dense 3D point cloud, where landslide-related features such as scarps, cracks, displaced material, and rupture boundaries appear as localized geometric discontinuities. These geometric irregularities form the fundamental signal exploited in this work for landslide-candidate detection.

Despite these sensing advances, many landslide detection and susceptibility approaches still rely on rasterized Digital Elevation Models (DEMs), 2D terrain derivatives, or image-based representations such as slope, curvature, roughness, multispectral imagery, or susceptibility covariates [3,8]. These representations are useful for regional-scale mapping, but they can suppress native 3D geometric structure and introduce slope-induced ambiguity. For example, steep but intact terrain may be assigned high instability scores because it resembles failed terrain in DEM-derived slope or roughness space, even when it lacks rupture boundaries, displaced material, or coherent scar morphology [8]. This limitation is especially important for rapid post-event mapping, where the relevant signal may be a localized 3D surface disruption rather than a broad regional susceptibility pattern.

Machine learning and hybrid statistical–machine learning methods have improved landslide susceptibility prediction by learning nonlinear relationships among terrain, hydrological, geological, and environmental variables [9,10,11]. Deep learning methods have also been applied to optical, multispectral, and DEM-derived landslide mapping, including unsupervised or weakly supervised feature-learning pipelines [12]. However, these methods commonly require labeled inventories, curated covariates, pre-defined raster products, or region-specific training data. Their performance may therefore degrade when transferred to new terrain types, post-disaster conditions, or areas where labeled landslide inventories and pre-event baselines are unavailable [9,12].

In contrast, 3D geometric morphometry computed directly from point clouds provides physically interpretable indicators of terrain instability. Local plane residuals, eigenvalue-based curvature, surface roughness, height discontinuity, and deviations from locally fitted surfaces can capture intrinsic structural disruptions associated with scarps, fractured surfaces, displaced blocks, and debris-like terrain. Point-cloud and terrestrial laser scanning (TLS) studies have demonstrated the value of 3D geometric analysis for deformation monitoring, rockfall detection, and dense displacement estimation [13,14,15]. However, many existing 3D workflows rely on multi-epoch change detection, terrestrial sensing infrastructure, hazard-specific pipelines, or supervised point-cloud models, which limits their suitability for single-survey, rapid UAV-based landslide-candidate mapping [13,14,15].

We posit that landslide-candidate regions can be characterized as structured geometric violations of local surface consistency in 3D space. Instead of relying on rasterized terrain abstractions, historical inventories, or data-driven supervision, landslide scar detection can be formulated as a geometry-driven inference problem in which instability emerges from multi-scale deviations in native point-cloud structure. This is visually illustrated in Figure 1, where landslide-related regions appear as localized departures from otherwise continuous terrain geometry.

To operationalize this idea, we propose *GeoFusion–3D*, a fully unsupervised framework for 3D landslide-candidate patch detection and post-failure susceptibility support from UAV-mounted LiDAR point clouds. The framework:

1. reduces slope-induced false positives through PCA-based local plane residual modeling;
2. enforces volumetric coherence of geomorphically anomalous regions through density-based 3D clustering; and
3. constructs a robust probabilistic instability score using IQR-normalized fusion of curvature, slope deviation, depth anomaly, height variation, and surface roughness.

The resulting system enables rapid, zero-shot landslide-candidate detection directly from native 3D point-cloud geometry, without requiring labeled training data, pre-event baselines, temporal differencing, or DEM rasterization. Importantly, the contribution of this work is not the invention of fundamentally new geometric operators or clustering algorithms. Rather, the novelty lies in reformulating landslide scar detection as a geometry-first inference problem on native 3D point clouds and integrating established geometric and statistical components into a unified unsupervised framework tailored for post-failure terrain analysis. By combining local surface modeling, volumetric anomaly aggregation, robust multi-feature fusion, and spatially coherent region growing, GeoFusion–3D provides an interpretable alternative to raster- or supervision-dependent approaches for rapid landslide-candidate mapping in complex terrain.

## 2. Related Work

Building on the limitations discussed in the previous section, we now review existing approaches to landslide detection and susceptibility modeling, focusing on their underlying assumptions and limitations with respect to rapid, geometry-driven inference from raw 3D data. Existing studies can broadly be grouped into three categories: (1) image- and DEM-based susceptibility mapping methods, including both supervised and unsupervised learning approaches; (2) multi-epoch deformation or change-detection frameworks that infer instability through temporal differencing; and (3) native point-cloud geometric analysis methods that operate directly on 3D spatial structure. While substantial progress has been made in the first two categories, the third remains comparatively underexplored, particularly in the context of single-survey, zero-shot landslide scar inference from UAV-acquired point clouds.

### 2.1. Statistical Landslide Susceptibility Modeling

Statistical methods remain among the most widely adopted approaches for landslide susceptibility analysis. Reichenbach et al. [3] conducted a comprehensive meta-analysis of over 560 studies, identifying more than 160 statistical and data-driven models. Their work highlights persistent challenges, including scale inconsistencies, heterogeneous validation practices, and lack of standardized workflows. To address these issues, Rossi et al. [4] introduced the LAND-SUITE toolbox, which provides a unified framework for reproducible susceptibility mapping.

Recent work has extended these models toward dynamic formulations. Samia et al. [5] proposed path-dependent susceptibility, where prior landslide occurrences influence future failure probabilities. While such approaches improve temporal modeling, they remain fundamentally reliant on historical inventories and spatial aggregation, limiting their applicability in rapid, post-event scenarios.

### 2.2. Machine Learning and Hybrid Statistical–Machine Learning Frameworks

Machine learning (ML) approaches have been widely explored for landslide susceptibility mapping due to their ability to capture nonlinear relationships among geomorphic and environmental variables. For example, Sahin et al. [10] demonstrated that random forest models provide improved stability over logistic regression in post-seismic susceptibility mapping.

Hybrid approaches further enhance predictive performance by combining multiple models. Li et al. [11] integrated neural networks, support vector machines, and random forests using a Grey Wolf Optimizer to generate composite susceptibility maps. Despite improved accuracy, these methods remain dependent on curated feature sets and labeled data, limiting generalization across regions with differing geomorphic characteristics.

### 2.3. Unsupervised Classification of LiDAR-Derived Terrain Features

Unsupervised methods have been applied to LiDAR-derived terrain features, typically extracted from Digital Elevation Models (DEMs). Abad et al. [8] demonstrated that clustering techniques such as k-means and Gaussian Mixture Models can achieve up to 87% classification accuracy in identifying landslide-affected terrain.

However, these approaches operate on rasterized representations and rely on 2D features such as slope and roughness. As discussed in Section 1, such abstractions inherently discard 3D structural information and are susceptible to slope-induced bias, often failing to distinguish steep but stable terrain from actual failure zones.

### 2.4. Deep Learning and Unsupervised Feature Learning

Deep learning approaches have also been extensively applied to landslide detection, particularly using optical and multispectral imagery. Tang et al. [12] employed convolutional autoencoders to learn latent representations of terrain features, followed by clustering for landslide identification. Their results demonstrate the benefits of learned feature representations over handcrafted descriptors.

Nevertheless, these methods remain data-dependent and require large labeled or semi-labeled datasets. Moreover, their reliance on spectral and raster-based inputs makes them sensitive to illumination variability, vegetation cover, and sensor artifacts. As a result, their performance often degrades in real-world, heterogeneous environments and post-disaster conditions where labeled data is unavailable.

### 2.5. 3-D LiDAR and Point-Cloud-Based Hazard Detection

Direct analysis of 3D point clouds offers a more principled representation of terrain structure. Blanco et al. [14] demonstrated the effectiveness of using geometric features derived from LiDAR point clouds for rockfall detection. Similarly, Wang et al. [15] proposed a hierarchical fusion framework combining terrestrial laser scanning (TLS) data with RGB imagery to estimate dense 3D displacement fields.

While these approaches highlight the potential of 3D geometric analysis, most existing LiDAR-based methods focus either on multi-epoch deformation estimation, infrastructure-intensive sensing pipelines, or hazard-specific applications such as rockfall analysis. Consequently, their operational scalability for rapid, large-area UAV deployment remains limited. Comparatively fewer studies investigate single-survey landslide scar detection directly from raw UAV point clouds using geometry-driven anomaly inference without temporal differencing or labeled supervision.

### 2.6. Geostatistical and Trigger-Based Spatial Modeling

Geostatistical approaches model spatial dependencies in landslide occurrence. Lombardo et al. [16] used spatial random effect models to capture residual spatial patterns in earthquake-triggered landslides. Such methods provide valuable insights into triggering mechanisms and spatial correlations.

However, these frameworks operate on aggregated spatial grids and do not explicitly model the fine-scale geometric structure of terrain deformation. Consequently, they are not well suited for detecting localized surface disruptions directly from raw 3D data.

### 2.7. Limitations of Existing Approaches

Despite substantial progress, existing landslide detection methods exhibit several fundamental limitations that hinder their applicability in rapid, zero-shot settings:

- **Reliance on 2D terrain abstractions.** Most approaches operate on DEM-derived features, which inherently discard 3D structural information and introduce slope-induced bias. This leads to frequent misclassification of steep but stable terrain.
- **Dependence on labeled data and historical inventories.** Supervised and hybrid learning methods require extensive, region-specific training data, limiting their ability to generalize across diverse terrains and post-disaster scenarios.
- **Underutilization of native 3D geometry.** Although point clouds contain rich structural information, most methods reduce them to low-dimensional representations. Fully exploiting geometric descriptors such as local plane residuals, curvature, and anisotropy remains largely unexplored for landslide detection.
- **Limited rapid and operational scalability.** High-precision systems such as TLS provide accurate measurements but are constrained by slow acquisition cycles and logistical complexity, preventing rapid deployment.
- **Lack of zero-shot, single-survey capability.** Existing approaches typically require pre-event data, temporal comparisons, or labeled inventories, none of which are guaranteed immediately after a disaster.

**Our Contribution.** To address these limitations, we introduce a fully unsupervised framework for landslide patch detection that operates directly on native 3D point clouds acquired from UAV LiDAR surveys. Unlike prior approaches that rely on rasterized terrain products, temporal differencing, or labeled inventories, our method formulates landslide detection as a geometry-driven inference problem based on multi-scale structural anomalies in surface geometry. Specifically, we (1) eliminate slope-induced bias using PCA-based local plane residuals computed in native 3D space, (2) enforce volumetric coherence through density-based clustering, and (3) construct a robust geomorphic anomaly score via IQR-normalized multi-feature fusion.

Crucially, the proposed approach operates in a zero-shot setting, requiring no labeled data, pre-event imagery, or historical inventories. This enables scalable, rapid landslide detection and susceptibility assessment directly from UAV-acquired LiDAR data, making it well suited for rapid post-disaster response and operational hazard monitoring.

## 3. Methodology

**Figure 2:**
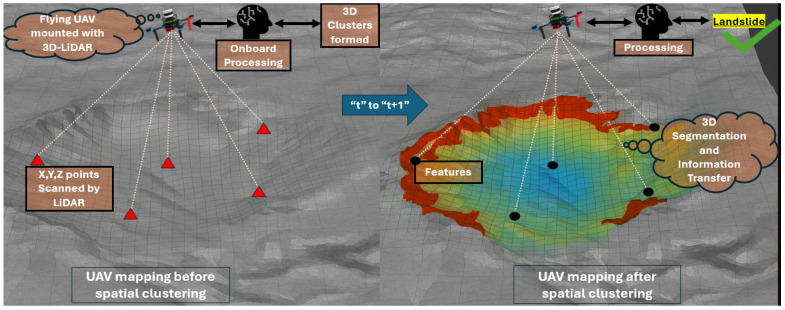
Overview of the proposed unsupervised zero-shot framework for identifying landslide-prone terrain using UAV-mounted 3D LiDAR. Phase 1 (**left**) illustrates raw LiDAR acquisition at time *t*, where XYZ points are collected but remain unstructured. Phase 2 (**right**) depicts the subsequent processing at time t+1, including spatial clustering, geometric feature extraction, and anomaly-driven segmentation to isolate unstable terrain regions with three colors of red (stable terrain), yellow (weak instability), green (moderate instability), and blue (high instability).

**Figure 3:**
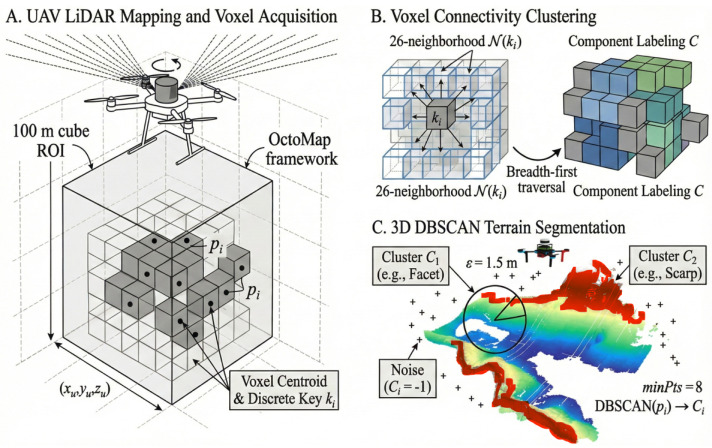
Voxel-level pre-segmentation pipeline. The UAV-mounted LiDAR produces a continuously updated OctoMap, from which voxel centroids, discrete keys, and coarse connected components are extracted prior to point-level processing.

**Figure 4:**
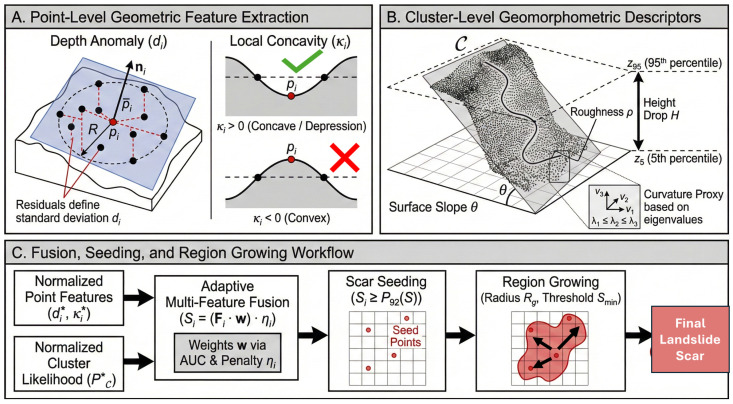
Pipeline for point-level geometric feature extraction, cluster-scale geomorphometric descriptor computation, and adaptive multi-feature fusion yielding the final point-level landslide confidence score.

This section presents the GeoFusion–3D pipeline for zero-shot landslide-candidate detection from UAV-mounted LiDAR point clouds. The method assumes that landslide-like regions appear as structured geometric deviations from locally consistent terrain surfaces. As shown in Figure 2, GeoFusion–3D first organizes raw XYZ measurements into a voxel-based volumetric representation, then extracts multi-scale geometric descriptors and fuses them into a final instability-confidence field. The inference procedure consists of four main steps. First, occupied voxels are extracted within a local region of interest and grouped using 26-neighborhood connectivity. Second, the structured point cloud is segmented using 3D DBSCAN to form coherent geomorphic clusters. Third, point-level features, including local plane residual depth and concavity, are combined with cluster-level descriptors, including slope, curvature proxy, roughness, height drop, and cluster depth anomaly. Finally, the normalized features are fused into a confidence score, high-confidence seeds are selected from the upper tail of the score distribution, and region growing produces spatially coherent landslide-candidate patches. Figure 3 and Figure 4 provide the detailed visual breakdown of these stages.

### 3.1. Phase 1: UAV LiDAR Mapping and Pre-Segmentation

Phase 1 converts raw airborne LiDAR returns into a structured spatial representation suitable for later geometric analysis. Its main purpose is to reduce noise, preserve local connectivity, and generate coarse terrain components before fine-grained landslide reasoning is performed. As illustrated in Figure 3, this stage consists of three sequential operations: voxel acquisition from the onboard OctoMap, voxel-connectivity grouping, and density-based terrain segmentation.

#### 3.1.1. UAV LiDAR Mapping and Voxel Acquisition

The first component of Phase 1 is shown in Figure 3A. The UAV carries a rotating 3D LiDAR sensor and incrementally maintains a probabilistic volumetric map using the OctoMap framework [17]. Rather than processing the full global map at each cycle, we extract a local cubic region of interest centered at the UAV position $\left(x_{u},y_{u},z_{u}\right)$. This region is defined as

(1) $$V=\left\{\left(x,y,z\right)\in \mathrm{OctoMap}\;|\;∥\left(x,y,z\right)-\left(x_{u},y_{u},z_{u}\right){∥}_{\infty }\leq 50\;m\right\}.$$

Equation (1) defines a 100 m cube around the UAV using the $\ell_{\infty }$ norm, which is computationally convenient for axis-aligned voxel maps. In practice, this local extraction bounds memory and computation while retaining enough spatial context for terrain analysis. The side length can be adjusted depending on terrain extent and onboard compute resources.

Each occupied OctoMap leaf node within V is then converted into a voxel centroid

(2) $$p_{i}=\left(x_{i},y_{i},z_{i}\right),\;i=1,\ldots ,N,$$

where *N* is the number of occupied voxels in the local region. Equation (2) provides the basic 3D point representation used throughout the remainder of the pipeline.

To support efficient indexing and connectivity reasoning, each centroid is further assigned a discrete spatial key

(3) $$k_{i}=\left(\left\lfloor \frac{x_{i}}{r}\right\rfloor ,\;\left\lfloor \frac{y_{i}}{r}\right\rfloor ,\;\left\lfloor \frac{z_{i}}{r}\right\rfloor \right),$$

where *r* is the OctoMap resolution. Equation (3) maps each continuous centroid location to an integer lattice coordinate. This discrete keying scheme is important because it enables hash-based storage, constant-time lookup, and efficient neighborhood traversal during the connectivity stage shown in Figure 3B.

#### 3.1.2. Voxel Connectivity Clustering

After voxelization, isolated occupied cells may still arise from sensor noise, sparse returns, or transient artifacts. To impose coarse spatial coherence, we group neighboring voxels using a Chebyshev-distance-based 26-neighborhood adjacency rule:

(4) $$k_{j}\in N\left(k_{i}\right)\;\Leftrightarrow \;{∥k_{j}-k_{i}∥}_{\infty }\leq 1.$$

Equation (4) defines voxel adjacency under the $\ell_{\infty }$ (Chebyshev) norm in a discrete 3D lattice, commonly referred to as 26-neighborhood connectivity. Two voxels are considered adjacent if their grid indices differ by at most one along each axis, thereby including face-, edge-, and corner-adjacent neighbors.

The use of 26-neighborhood connectivity is deliberate: unlike 6-neighborhood (face-only) or 18-neighborhood (face + edge), it captures full volumetric connectivity, ensuring that diagonally connected structures are not artificially fragmented. This is particularly important for natural terrain, where surfaces such as slopes, scarps, and debris flows exhibit continuous but non-axis-aligned geometry. By incorporating corner adjacency, the formulation preserves geometric coherence across irregular structures while maintaining consistency with the underlying cubic voxel lattice.

Using this adjacency graph, a breadth-first traversal yields a component-labeling map

(5) $$C:\left\{1,\ldots ,N\right\}\to Z_{\geq 0},$$

which assigns each voxel to a connected component. Equation (5) does not yet identify landslides; instead, it performs structural regularization by separating coherent terrain masses from isolated noise. This step corresponds to the component-labeling block in Figure 3B and prepares the point cloud for finer terrain segmentation.

#### 3.1.3. 3D DBSCAN Terrain Segmentation

The final step of Phase 1 is shown in Figure 3C. After multiple UAV passes, the accumulated point cloud becomes dense enough to support density-based clustering of natural terrain structures. We apply 3D-DBSCAN [18] with

(6) ε=1.5m,minPts=8,

and assign cluster labels through

(7) $$\mathrm{DBSCAN}\left(p_{i};\varepsilon ,\mathrm{minPts}\right)\to C_{i}.$$

The choice of ε=1.5m is guided by the spatial resolution of the voxelized LiDAR representation and the expected geometric scale of terrain features. Specifically, ε is set to be slightly larger than the voxel resolution to ensure that neighboring surface points belonging to the same physical structure remain connected, while still being small enough to avoid merging distinct terrain elements such as adjacent slope faces or discontinuities.

The parameter minPts=8 is selected to enforce a minimum local support consistent with 3D volumetric neighborhoods. In particular, it approximates the minimum number of points required to form a stable local surface patch under noisy LiDAR sampling, while rejecting sparse or transient returns. This value balances robustness to noise with sensitivity to fine-scale geomorphic structures.

Equation (6) therefore defines the density threshold for cluster formation, while Equation (7) assigns a cluster label $C_{i}$ to each point $p_{i}$. The resulting clusters correspond to coherent geomorphic units such as slope facets, benches, scarps, and displaced deposits. Points failing the density criterion are labeled as noise ($C_{i}=-1$, in white color). This density-based segmentation is particularly appropriate for natural terrain because it preserves irregular boundaries while suppressing isolated outliers, as visually shown in Figure 3C.

Taken together, Equations (1)–(7) convert unstructured LiDAR returns into a denoised, spatially organized terrain representation, which serves as the input to Phase 2 for landslide-specific geometric reasoning.

### 3.2. Phase 2: Geometric Feature Extraction and Multi-Scale Confidence Fusion

Phase 2 transforms the structured point cloud from Phase 1 into a hierarchy of interpretable geometric descriptors. The central goal is to combine fine-grained point-level evidence of local disruption with cluster-level evidence of broader geomorphic instability. Figure 4 summarizes this logic in three parts: point-level feature extraction (panel A), cluster-level geomorphometric characterization (panel B), and fusion, seeding, and region growing (panel C).

Formally, Phase 1 supplies (i) a denoised point set $P=\{p_{i}\}$ and (ii) a set of DBSCAN clusters $\left\{C_{1},C_{2},\ldots \right\}$. For neighborhood-based operations, we consider the planar distance

(8) $$d_{\epsilon }\left(i,j\right)=∥\left(x_{i},y_{i}\right)-\left(x_{j},y_{j}\right)∥,$$

which measures horizontal proximity between points. Equation (8) is used because many landslide signatures, such as scar edges and localized depressions, are best interpreted relative to nearby terrain on the surface plane rather than by full 3D Euclidean distance, which may mix vertical relief with lateral support.

#### 3.2.1. Point-Level Depth Anomaly

The first point-level descriptor is the depth anomaly shown in Figure 4A (left), where a local plane is fit to the neighborhood of each point and deviations from that plane are measured. For each point $p_{i}$, we define a planar neighborhood as

(9) $$N_{i}=\;\{p_{j}:∥\left(x_{j},y_{j}\right)-\left(x_{i},y_{i}\right)∥\;\leq R\},$$

where *R* is the neighborhood radius. Equation (9) determines the local support used to estimate the underlying terrain surface around $p_{i}$.

Given $N_{i}$, we compute the local centroid

(10) $${\bar{p}}_{i}=\frac{1}{|N_{i}|}\sum_{j\in N_{i}}p_{j},$$

and covariance matrix

(11) $$C_{i}=\mathrm{Cov}\left(p_{j}-{\bar{p}}_{i}\right).$$

Equation (10) defines the local mean position, while Equation (11) captures the orientation and spread of the neighborhood. The smallest-eigenvalue eigenvector of $C_{i}$ gives the local surface normal $n_{i}$ [19], since it corresponds to the direction of least variation.

We then define the depth anomaly as the root-mean-square orthogonal residual to this local plane:

(12) $$d_{i}=\sqrt{\frac{1}{|N_{i}|}\sum_{j\in N_{i}}{\left(n_{i}^{⊤}\left(p_{j}-{\bar{p}}_{i}\right)\right)}^{2}}.$$

Equation (12) is a central quantity in the proposed method. It measures how strongly a neighborhood departs from local planarity. Small values indicate smooth, surface-consistent terrain; large values indicate geometric disruption, such as cracks, scarps, fractured surfaces, or displaced debris. This is why the residual diagram in Figure 4A directly reflects the type of discontinuity expected in active landslide regions.

#### 3.2.2. Point-Level Local Concavity

Depth anomaly captures local roughness and discontinuity, but it does not explicitly distinguish whether a point lies in a locally depleted region. For this reason, we introduce a second point-level descriptor: local concavity, shown in Figure 4A (right). It is defined as

(13) $$\kappa_{i}=\left(\frac{1}{|N_{i}|}\sum_{j\in N_{i}}z_{j}\right)-z_{i}.$$

Equation (13) compares the elevation of a point to the mean elevation of its neighborhood. Positive $\kappa_{i}$ implies that the point lies below its local surroundings, indicating a concave or depressed configuration that is often associated with depletion zones, collapse pockets, or gravitational subsidence. Negative values instead indicate convex surface positions. In Figure 4A, this behavior is illustrated schematically: concave depressions are retained as instability evidence, whereas convex protrusions are not treated the same way.

Together, Equations (12) and (13) define the two main point-level signals used throughout the remainder of the method.

#### 3.2.3. Cluster-Level Geomorphometric Descriptors

Point-level features alone are often too local to robustly characterize an entire landslide patch. Therefore, for each DBSCAN cluster C, we compute a set of cluster-level geomorphometric descriptors, illustrated in Figure 4B.

##### Surface Slope

For each cluster, we fit a local plane z=ax+by+c and define the representative slope angle as

(14) $$\theta =\tan^{-1}\;\left(\sqrt{a^{2}+b^{2}}\right).$$

Equation (14) summarizes the overall inclination of the cluster. Larger values indicate steeper terrain, which is often more susceptible to mass wasting.

##### Curvature Proxy

Let $\lambda_{1}\leq \lambda_{2}\leq \lambda_{3}$ be the eigenvalues of the cluster covariance structure. We define a curvature proxy as

(15) $$\mathrm{curv}=\frac{\lambda_{1}}{\lambda_{1}+\lambda_{2}+\lambda_{3}}.$$

Equation (15) measures how strongly the cluster departs from a planar configuration. When $\lambda_{1}$ is very small relative to the total variance, the surface is approximately planar; larger ratios indicate stronger curvature concentration and more irregular local structure.

##### Surface Roughness

We define roughness as the mean absolute vertical variation within the cluster:

(16) $$\rho =\frac{1}{|C|-1}\sum_{i}\left|z_{i+1}-z_{i}\right|.$$

Equation (16) captures local vertical variability and serves as a coarse indicator of fragmented or debris-like terrain. In practice, higher roughness values often correspond to disturbed deposits or fractured surfaces.

##### Height Drop

To represent vertical relief while remaining robust to outliers, we define the cluster height drop as

(17) $$H=z_{95}-z_{5},$$

where $z_{5}$ and $z_{95}$ are the 5th and 95th percentile elevations in the cluster. Equation (17) reflects the vertical span of the cluster and is visually represented in Figure 4B. Large values are characteristic of scarps, escarpments, or vertically displaced masses.

##### Cluster Depth Anomaly

Finally, we aggregate point-level residual structure over the full cluster:

(18) $$D_{C}=\sqrt{\frac{1}{\left|C\right|}\sum_{i\in C}r_{i}^{2}}.$$

Equation (18) measures the average depth-irregularity magnitude across the cluster, where $r_{i}$ denotes the local residual associated with point *i*. This provides a cluster-scale analog of the point-level depth anomaly in Equation (12).

Taken together, Equations (14)–(18) encode the broader geomorphic character of each terrain unit. This is the cluster-scale evidence shown in Figure 4B and later used to construct the initial landslide-confidence score.

### 3.3. Cluster-Level Landslide Confidence Score

Because the descriptors in Equations (14)–(18) are measured on different scales, they must first be normalized before meaningful fusion. We adopt a robust median–IQR logistic transformation:

(19) $$f^{★}=\sigma \;\left(\frac{f-\mathrm{median}(f)}{\mathrm{IQR}(f)}\right),\;\sigma \left(x\right)=\frac{1}{1+e^{-x}}.$$

Equation (19) centers each feature by its median, scales it by the interquartile range, and then maps it through a logistic function. This is important because geomorphic variables are often heavy-tailed and can vary significantly across sites. The transformation yields comparable, bounded feature values while remaining robust to outliers.

Using these normalized descriptors, we define the cluster-level landslide-confidence score as

(20) $$P_{C}=w_{\theta }\;\theta^{★}+w_{\mathrm{curv}}\;{\mathrm{curv}}^{★}+w_{D}\;D_{C}^{★}+w_{\rho }\;\rho^{★}+w_{H}\;H^{★}.$$

Equation (20) combines slope, curvature concentration, depth irregularity, roughness, and vertical relief into a single measure of geomorphic instability. Intuitively, high values of $P_{C}$ indicate terrain units whose global structure is consistent with landslide morphology. In the workflow of Figure 4C, this score forms the cluster-level likelihood term that is later fused with point-level evidence.

Each point $p_{i}\in C$ inherits the cluster score $P_{C}$. For DBSCAN noise points, which do not belong to any coherent terrain unit and therefore lack cluster context, we define a fallback score using only normalized depth anomaly:

(21) $$P_{i}=0.15+0.85\;d_{i}^{★}.$$

Equation (21) ensures that isolated but strongly irregular points are not discarded entirely, while still giving primary emphasis to structured cluster-level evidence.

For interpretability, the continuous per-point score $P_{i}$ is mapped to four qualitative classes used throughout the experiments:

- **Red ($P_{i}<0.50$)**: morphologically stable terrain with no meaningful surface disruption;
- **Yellow ($0.50\leq P_{i}<0.60$)**: weak or incipient instability;
- **Green ($0.60\leq P_{i}<0.70$)**: moderate landslide activity with partial geomorphic coherence;
- **Blue ($P_{i}\geq 0.70$)**: strong instability, typically associated with active scarps, failed masses, or debris-dominated terrain.

This categorical interpretation is not used for training or supervision; rather, it provides a human-readable view of the continuous instability field derived from Equation (20).

### 3.4. Adaptive Multi-Feature Fusion

Although the cluster score captures broader terrain organization, landslides also exhibit fine-scale pointwise variations that should not be ignored. Therefore, as illustrated in Figure 4C, we combine cluster-scale confidence with the normalized point-level signals into a compact feature vector:

(22) $$F_{i}=\left[\begin{array}{c} P_{i},\;d_{i}^{★},\;\kappa_{i}^{★} \end{array}\right].$$

Equation (22) brings together three complementary signals: inherited cluster likelihood, local surface discontinuity, and local depression geometry. Their combination allows the model to distinguish broad unstable regions from isolated noisy fluctuations.

The fusion weights w are determined using an AUC-maximizing ranking procedure based on pseudo-labels extracted from the upper tail of the instability distribution. To ensure that the inherited geomorphic likelihood remains influential, we impose the constraint

(23) $$w_{1}\geq 0.3.$$

Equation (23) prevents the fusion model from overemphasizing purely local signals at the expense of cluster-scale context. The final fused point-level confidence score is then

(24) $$S_{i}=\left(F_{i}\cdot w\right)\cdot \eta_{i},$$

where $\eta_{i}$ is a stability-aware penalty defined as

(25) $$\eta_{i}=\left\{\begin{array}{cc} 0.3, & d_{i}^{★}<0.2\;\mathrm{and}\;\kappa_{i}^{★}<0.2,\\ 1.0, & \mathrm{otherwise}. \end{array}\right.$$

Equation (24) performs the actual multi-feature fusion, while Equation (25) suppresses points that are simultaneously smooth and non-concave. This is important because such points are unlikely to correspond to genuine landslide structure even if they are near unstable regions. In Figure 4C, this corresponds to the adaptive fusion block preceding scar seeding.

#### Discrimination Between Rough Terrain and Landslide-like Instability

A key design choice is that roughness alone is insufficient to produce a high-confidence landslide-candidate label. Local roughness may occur in stable rocky terrain, vegetation-affected point clouds, gullies, or noisy resampled surfaces. GeoFusion–3D therefore requires multi-feature agreement across scales. A point or cluster receives high confidence only when roughness co-occurs with local plane residuals, positive concavity or depletion structure, curvature concentration, height drop, and spatially coherent cluster support. Isolated rough points are suppressed by DBSCAN noise rejection and by region growing, while steep but smooth terrain tends to have low residual depth and weak concavity despite high slope. This prevents the method from labeling every uneven surface as unstable.

### 3.5. Scar Seeding and Region Growing

The final stage converts the fused confidence map into explicit landslide patches. Rather than thresholding all points directly, we first identify high-confidence seed points from the upper tail of the fused score distribution:

(26) $$S=\left\{i\;|\;S_{i}\geq P_{92}\left(S\right)\right\},$$

where $P_{92}\left(S\right)$ denotes the 92nd percentile of the fused scores. Equation (26) selects the top 8% of points as candidate landslide seeds, corresponding to the scar-seeding step shown in Figure 4C. This percentile-based strategy is useful in zero-shot settings because no fixed site-specific threshold is assumed.

Seed points are then grouped using 2D DBSCAN in the horizontal plane, which preserves spatial coherence while reducing computational cost. Each seed cluster is subsequently expanded through radius-based region growing:

(27) $$j\in \mathrm{grow}\left(i\right)\;⟺\;∥\left(x_{j},y_{j}\right)-\left(x_{i},y_{i}\right)∥\leq R_{g}\;\;\mathrm{and}\;\;S_{j}\geq S_{\min}.$$

Equation (27) states that a point *j* is absorbed into the growing scar region of seed *i* if it lies within planar radius $R_{g}$ and maintains at least a minimum confidence $S_{\min}$. This allows high-confidence seed regions to expand into adjacent points that preserve local geometric consistency, yielding spatially coherent scar boundaries rather than isolated detections.

Clusters below a minimum size threshold are removed, and the remaining grown regions constitute the final extracted landslide scars. This final output corresponds to the rightmost block in Figure 4C and completes the end-to-end mapping process summarized earlier in Figure 2.

The proposed methodology converts raw UAV LiDAR measurements into a structured instability field that reflects both local geometric disruption and broader geomorphic coherence [20,21]. By combining voxel-level organization, density-based terrain segmentation, point-scale anomaly estimation, and cluster-aware confidence fusion, the framework produces spatially coherent landslide-scar candidates without supervision or pre-event reference data. This representation forms the basis for the empirical study that follows. In the next section, we evaluate the method across diverse terrains and controlled deformation scenarios to examine its accuracy, robustness, and cross-regional behavior.

## 4. Experiments

### 4.1. Dataset Provenance, Validation Design, and Experimental Setup

To avoid ambiguity, we distinguish the intended sensing modality from the validation data used in this study. GeoFusion–3D is designed for UAV-mounted LiDAR point clouds, but the controlled validation experiments in this manuscript use terrain-derived point clouds generated from high-resolution 3D terrain meshes. Specifically, high-resolution terrain meshes are obtained from Google Maps 3D tiles, imported into Blender, modified where synthetic landslide deformation is required, and then resampled into LiDAR-like point clouds with simulated range noise, beam divergence, and sparsity [22,23].

This validation strategy was adopted because openly accessible real UAV-LiDAR point-cloud datasets for the selected event settings, with sufficient point-level access, consistent metadata, and usable landslide-scar reference information, were not available to us. Rather than relying on incomplete or non-reproducible field data, we constructed controlled terrain-derived point clouds that preserve realistic topographic structure while allowing the location, shape, and severity of scar-like deformation to be known. This makes it possible to evaluate whether GeoFusion–3D can recover landslide-like deformation patterns under realistic terrain geometry and LiDAR-like sampling artifacts, without claiming that all validation data were collected from physical UAV flights.

We evaluate the proposed GeoFusion–3D framework under diverse geomorphic conditions to assess its ability to detect landslide-like scar regions in a fully unsupervised, zero-shot setting. The experimental design is structured around three core objectives:

1. **Cross-regional generalization:** Evaluate performance across terrains with distinct geomorphic characteristics.
2. **Robustness to deformation and noise:** Assess detection under controlled synthetic landslide scenarios and LiDAR-like sampling artifacts.
3. **False-positive suppression:** Measure behavior in low-susceptibility terrain where over-detection is most likely.

Because the proposed framework does not rely on labeled training data, pre-event baselines, or historical inventories, the validation design must explicitly separate three different forms of evidence: controlled synthetic deformation, negative-control terrain, and qualitative cross-regional geomorphic interpretation. The controlled synthetic cases provide known deformation targets for evaluating detection behavior. The negative-control case evaluates whether the method avoids over-detecting stable terrain. The cross-regional cases examine whether the same geometry-driven scoring highlights plausible landslide-like structures across different landscapes, but these detections are interpreted as candidate instability zones unless independently field validated.

To ensure broad coverage of geomorphic conditions, we select three representative sites spanning high-relief, coastal, and low-susceptibility environments:

1. **Washington Creek, North Cascades (WA)**—high-relief alpine terrain with steep slopes, sharp breaklines, and pronounced scarps. The North Cascades are characterized by significant vertical relief and steep slopes resulting from tectonic uplift and glacial incision [24,25]. Glacial erosion has produced deep valleys, sharp ridge-valley transitions, and rugged topography, while landslide processes generate unstable scarps and geomorphic discontinuities [26].
  *Reason for selection and validation role:* Washington Creek is selected as the high-relief benchmark because steep terrain, sharp topographic breaks, and complex surface morphology are challenging for geometry-based landslide detection. In this case, the terrain mesh is used as the base surface, and controlled Blender deformation is injected to create a scar-like target with known location and morphology. This provides a controlled validation case for testing whether GeoFusion–3D can recover pronounced scarp-like deformation, displaced material, and local height discontinuities while avoiding confusion with naturally steep but intact slopes.
2. **Kincaid Park, Anchorage (AK)**—coastal bluff terrain characterized by shallow rotational failures and marine erosion. Coastal bluff systems in Anchorage are widely documented to exhibit shallow landslides driven by marine erosion, freeze-thaw cycles, and soil saturation, resulting in rotational slope failures and progressive retreat of bluff faces [27,28].
  *Reason for selection and validation role:* Kincaid Park is selected as the subtle-deformation benchmark because coastal bluff failures often produce shallow, diffuse, and low-amplitude surface changes rather than sharply exposed scarps. The terrain mesh is therefore modified with controlled synthetic deformation representing shallow rotational or bluff-retreat-like instability. This case evaluates whether the method can detect weak concavity, residual-depth anomalies, and cluster-scale deformation coherence without treating all ordinary bluff roughness as landslide evidence.
3. **Cushetunk Mountain, New Jersey (NJ)**—a basaltic ridge with globally low landslide susceptibility, used to evaluate false-positive behavior. Basaltic ridge systems such as Cushetunk Mountain are structurally stable due to competent igneous lithology and relatively low slope-driven failure susceptibility, making them suitable benchmarks for evaluating false-positive suppression in landslide detection frameworks [3,29].
  *Reason for selection and validation role:* Cushetunk Mountain is selected as the negative-control site. Unlike the Washington Creek and Kincaid Park cases, no synthetic landslide deformation is injected. The objective is not to recover a known scar, but to test whether GeoFusion–3D suppresses false positives in stable terrain that may still contain rough surfaces, local slope changes, or natural topographic irregularity. This case is important because a useful zero-shot detector should not label every rough or uneven region as unstable.

Accordingly, the validation data are grouped into four categories: controlled synthetic deformation cases, negative-control stable terrain, LiDAR-like resampled point clouds, and cross-regional qualitative cases. Table 1 summarizes the primary validation roles rather than listing scanner-specific acquisition parameters, because the point clouds used in this study are not physical UAV flight logs.

Across all environments, the same point-cloud generation and voxelization protocol is used. Terrain surfaces are first obtained as high-resolution 3D terrain meshes, processed in Blender where synthetic deformation is required, and then converted into LiDAR-like point clouds for evaluation. The resulting point clouds are mesh-derived and simulation-resampled rather than physical UAV flight logs. After resampling, all environments are processed through the same ROS/Gazebo and CTU-MRS simulation pipeline, with occupied voxels represented using OctoMap-based volumetric mapping [17,30,31]. Consequently, acquisition variables tied to a physical scanner, such as flight altitude, scanner model, pulse rate, and beam divergence, are treated as simulated or not applicable in this study. The coordinates are quantized at approximately 0.4 m in *x*, *y*, and *z*, and we therefore report 0.4 m as the effective voxelized point-cloud spacing used consistently throughout all environments. This resolution is governed by the OctoMap/CTU-MRS simulation configuration rather than by a real UAV-LiDAR sensor specification.

This setup enables systematic evaluation across three complementary regimes:

- **High-curvature terrain** (Washington Creek),
- **Subtle deformation** (Kincaid Park),
- **Stable terrain** (Cushetunk Mountain).

In addition to these three primary validation regimes, we include cross-regional qualitative cases to examine whether the same unsupervised scoring behavior transfers to other geomorphic settings. These cases are not treated as field-confirmed landslide inventories. Instead, detections are interpreted as landslide-like geomorphic instability candidates when multiple indicators co-occur, including scarp-like or arcuate boundaries, localized height drop, displaced or roughened downslope material, curvature concentration, and spatially coherent cluster support.

No claim is made that the synthetic deformation cases replace field-validated landslide inventories. Rather, they provide controlled ground truth for evaluating whether the proposed geometric fusion pipeline can recover known scar-like deformation patterns. Similarly, the cross-regional cases are used to assess qualitative transferability of the geometry-driven score, while final landslide confirmation would require expert geomorphic interpretation, field evidence, existing inventories, or multi-temporal validation.

### 4.2. Synthetic Scar Generation

Because pre-event LiDAR is rarely available in UAV deployments, we generate physically plausible synthetic landslide deformations to enable controlled validation. This approach allows direct assessment of detection accuracy under known ground-truth conditions while preserving realistic terrain geometry.

The synthetic pipeline consists of three stages:

1. **Terrain extraction:** High-resolution terrain meshes are obtained from 3D Google Maps tiles and imported into Blender.
2. **Physically guided deformation:** We simulate landslide processes including:
  - headscarp retreat and slope collapse,
  - translational block displacement,
  - toe bulging and deposition zones.
3. **LiDAR resampling and noise modeling:** The modified mesh is resampled into a point cloud and augmented with realistic LiDAR artifacts, including range noise, beam divergence, and vegetation-induced sparsity.

This design provides:

- controlled variation in deformation magnitude and geometry,
- paired before–after datasets,
- robustness evaluation under noise,
- quantitative analysis of detection performance.

Synthetic deformations are applied to Washington Creek and Kincaid Park, while Cushetunk Mountain remains unmodified to evaluate real-world false-positive behavior.

### 4.3. Qualitative Visualization of Synthetic and Real Terrains

**Figure 5:**
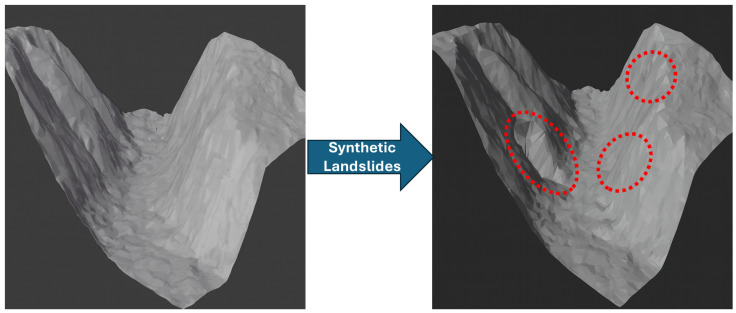
Synthetic deformation generated for the Washington Creek terrain, illustrating imposed scarps and displaced material encircled in red.

Figure 5 illustrates synthetic deformation applied to the Washington Creek terrain. The imposed landslide like regions introduce localized geometric discontinuities, including scarps and displaced mass, which directly correspond to the geometric signals (depth anomaly and concavity) described in Section 3.

**Figure 6:**
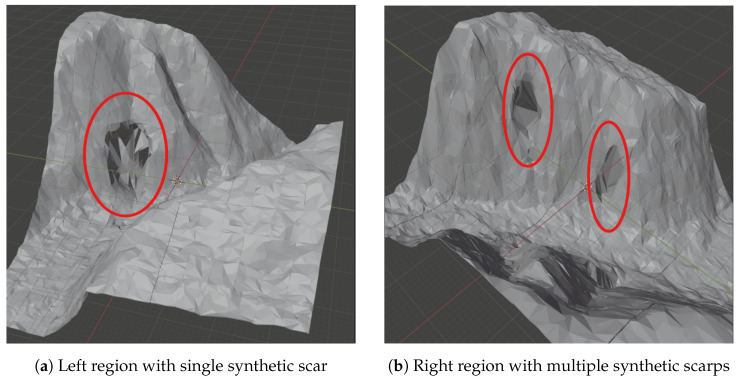
Localized views of Washington Creek after synthetic deformation as encircled in red.

Figure 6 shows localized regions of the Washington Creek dataset after deformation. The red-circled areas correspond to inserted landslide patches, which exhibit strong geometric discontinuities consistent with high depth-anomaly values (Equation (12)) and elevated cluster-level scores (Equation (20)).

**Figure 7:**
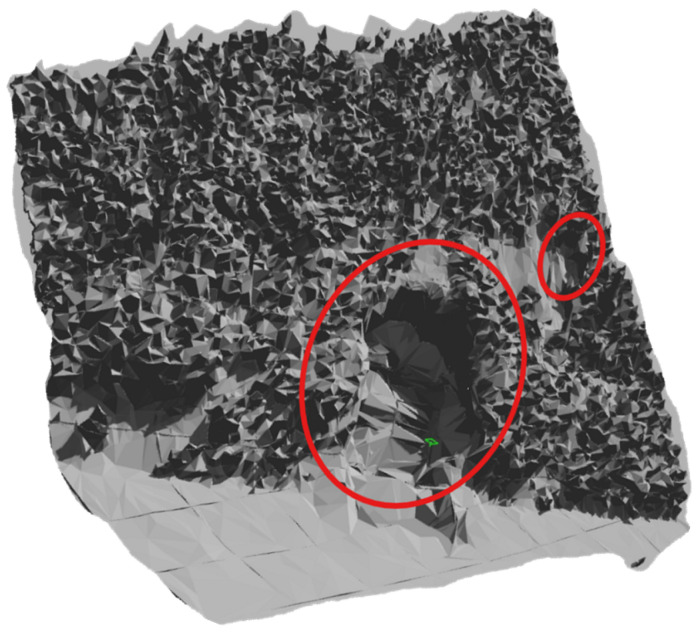
Synthetic deformation (encircled in red) applied to the Kincaid Park, Alaska coastal bluff terrain.

Figure 7 presents the synthetic deformation applied to the Kincaid Park coastal bluff terrain. Unlike the high-relief Washington Creek case, this site contains shallower and more spatially diffuse deformation superimposed on naturally uneven bluff morphology [32]. This setting is therefore used to test whether GeoFusion–3D can detect subtle shallow-failure patterns where the instability signal is weaker and less sharply bounded. In this case, detection relies on the combined response of local concavity (Equation (13)), residual depth anomaly (Equation (12)), and cluster-scale coherence, rather than roughness alone.

**Figure 8:**
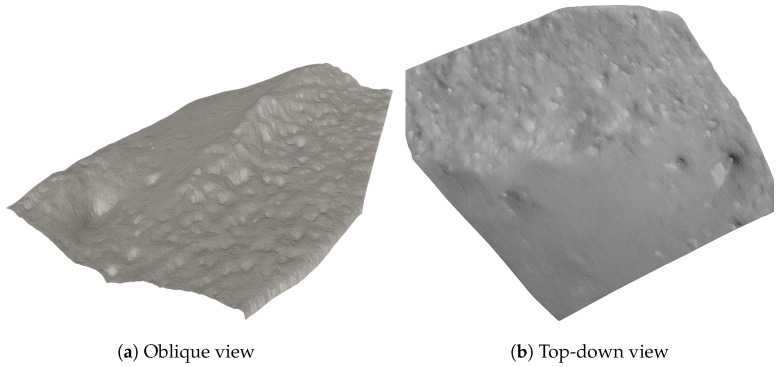
Cushetunk Mountain, NJ dataset used for false-positive evaluation.

Finally, Figure 8 shows the Cushetunk Mountain dataset, which remains unmodified. The absence of injected deformation allows evaluation of the system’s ability to suppress false positives in stable terrain where geometric discontinuities are minimal.

Collectively, these datasets span high-relief scarps, shallow rotational failures, synthetic deformations with known structure, and real stable terrain. This diversity ensures that GeoFusion–3D is evaluated across the full spectrum of geomorphic conditions relevant to operational landslide monitoring.

## 5. Results

We evaluate the proposed GeoFusion–3D framework across three primary validation regimes: (i) controlled synthetic deformation at Washington Creek (WA) and Kincaid Park (AK), (ii) the undeformed, low-susceptibility negative-control terrain of Cushetunk Mountain (NJ), and (iii) cross-regional qualitative cases used to assess transferability of the same geometry-driven scoring behavior. Table 2 provides a compact summary of the validation type, main evidence, and observed outcome for each case. The detailed results for each environment are then presented in the following subsections.

As summarized in Table 2, the selected cases collectively span high-relief alpine terrain, shallow coastal bluff morphology, stable ridge terrain, and additional cross-regional geomorphic settings. This design allows GeoFusion–3D to be evaluated across complementary regimes: recovery of known synthetic scar-like deformation, suppression of false positives in stable terrain, and qualitative transferability to landslide-like candidate regions in diverse landscapes.

Across these environments, GeoFusion–3D consistently highlights spatially coherent instability candidates rather than isolated rough points. The integration of point-level signals, including depth anomaly and concavity, with cluster-level descriptors, including curvature, slope, roughness, height drop, and cluster coherence, supports separation between intact terrain and landslide-like geomorphic anomaly zones. The following subsections provide the detailed case-by-case visual and quantitative interpretation underlying the summary in Table 2.

### 5.1. Synthetic Deformation Detection: Washington Creek

**Figure 9:**
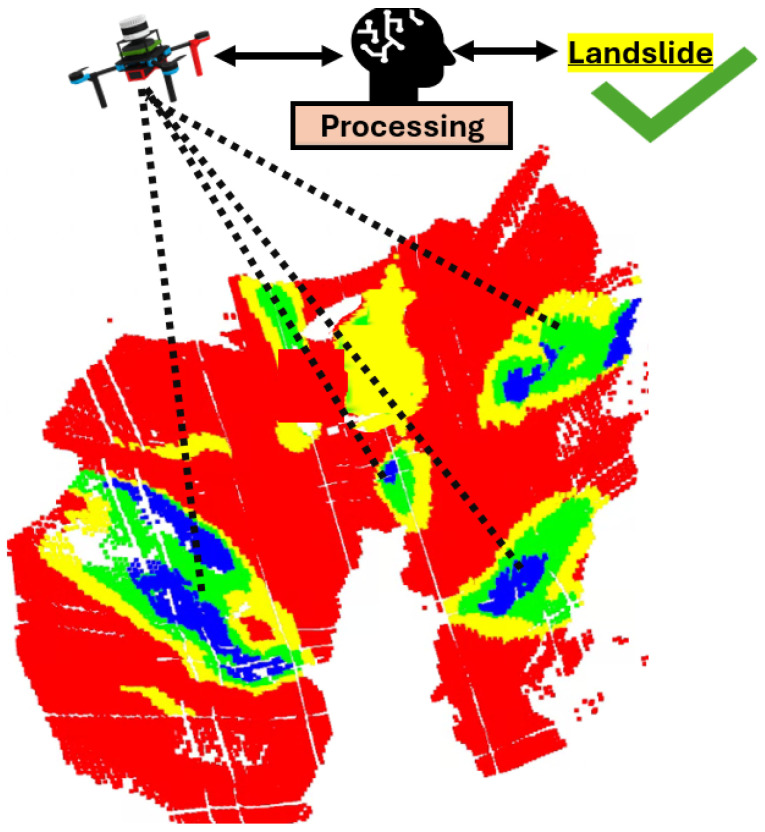
GeoFusion–3D instability mapping for synthetically deformed Washington Creek (Figure 6). Regions shown in blue and green indicate high and moderate instability, respectively, while red denotes stable terrain. At time t+1 (see Figure 2), the pipeline performs spatial clustering, geometric feature extraction, and anomaly-driven segmentation to isolate unstable terrain regions. The resulting semantic map encodes stability levels as follows: red (stable terrain), yellow (weak instability), green (moderate instability), blue (high instability), and white (noise).

Figure 9 shows the color-coded instability field generated for the synthetically deformed Washington Creek terrain. High-instability regions (blue/green) align with the injected landslide-like scars, while most surrounding stable terrain is classified as red. This result demonstrates that GeoFusion–3D does not rely on a single terrain descriptor, but instead identifies regions where several geometric indicators jointly support landslide-like deformation.

In GeoFusion–3D, concavity alone is not sufficient to produce a high-confidence landslide label. Natural valleys and gullies may exhibit positive concavity and height variation, but they often form broad, continuous drainage-like morphology with relatively smooth local surface structure. In contrast, the injected Washington Creek scar produces a localized combination of abrupt surface disruption, sharp relief change, curvature concentration, and spatially compact cluster coherence. Therefore, the model assigns high confidence only where concavity co-occurs with elevated local plane residuals (Equation (12)), curvature concentration (Equation (15)), height drop (Equation (17)), and coherent cluster-level support (Equation (20)).

At a larger spatial scale, Figure 9 illustrates the full-scene reconstruction and the extracted high-instability regions. The detected blue and green regions form spatially coherent patches over the imposed deformation zones rather than following every concave valley-like depression in the scene. This behavior is important because natural valleys, gullies, and canyon-like surfaces may contain concavity, but they do not necessarily contain the full multi-feature signature of a landslide scar.

For the Washington Creek result, the dominant prediction drivers are local plane residual, height drop, curvature concentration, and cluster-level coherence. The local plane residual is high along scar edges and displaced surfaces because these areas depart strongly from locally planar terrain. Height drop and curvature concentration further emphasize the abrupt scarp-like breaklines introduced by the synthetic deformation. Cluster-level coherence then suppresses isolated noisy responses and retains only spatially organized regions whose neighboring points share consistent instability evidence. Concavity contributes mainly in the depletion portions of the scar, but it is treated as supporting evidence rather than the sole decision variable. This explains why naturally concave terrain does not automatically become blue or green unless it also exhibits residual-depth disruption, curvature concentration, height discontinuity, and coherent cluster support.

Figure 9 also provides a finer-scale comparison between point-level anomaly detection and reconstructed surface geometry. The anomaly-based delineation closely follows the injected deformation geometry, indicating that local geometric signals (Equations (12) and (13)) capture true deformation patterns when they are reinforced by cluster-scale evidence. Yellow regions indicate weak or transitional instability responses, while high-confidence blue/green labels occur only where multiple descriptors agree.

This multi-feature behavior directly addresses the distinction between landslide scars and naturally concave landforms. A valley, gully, or canyon may be concave, but if it is geomorphically smooth and laterally continuous, it tends to have lower local plane residuals and weaker rupture-boundary curvature than a scar-like deformation. Conversely, a landslide scar is expected to produce a compact zone of disrupted local planarity, sharp elevation discontinuity, rough displaced material, and coherent cluster-scale anomaly. GeoFusion–3D therefore distinguishes landslide-like scars from ordinary concave terrain by requiring agreement across point-level residual structure, concavity, curvature, height drop, and cluster-level coherence rather than relying on concavity alone.

### 5.2. Sensitivity to Subtle Deformation: Kincaid Park

**Figure 10:**
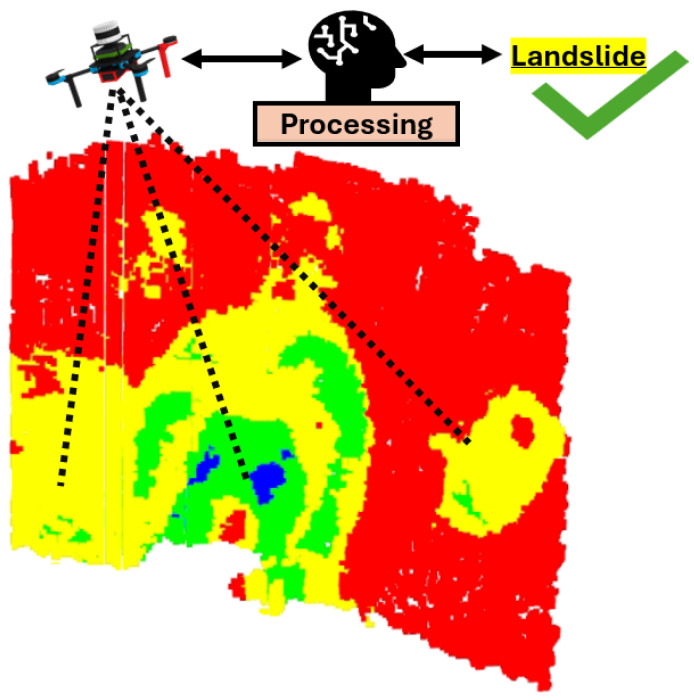
GeoFusion–3D instability mapping for synthetically deformed Kincaid Park terrain, Alaska (Figure 7). At time t+1 (see Figure 2), the framework performs spatial clustering, geometric feature extraction, and anomaly-driven segmentation to isolate unstable terrain regions. The resulting semantic map encodes stability levels as follows: red (stable terrain), yellow (weak instability), green (moderate instability), blue (high instability), white (noise).

Figure 10 presents results for the synthetically deformed Kincaid Park coastal bluff terrain. Unlike Washington Creek, deformation in this setting is lower in amplitude and more spatially diffuse.

Despite these challenges, GeoFusion–3D successfully identifies shallow failure zones, indicating strong sensitivity to subtle concavity-driven signals (Equation (13)). This demonstrates that the method is not limited to high-relief environments, but can also capture early-stage or low-magnitude deformation.

### 5.3. False-Positive Suppression: Cushetunk Mountain

**Figure 11:**
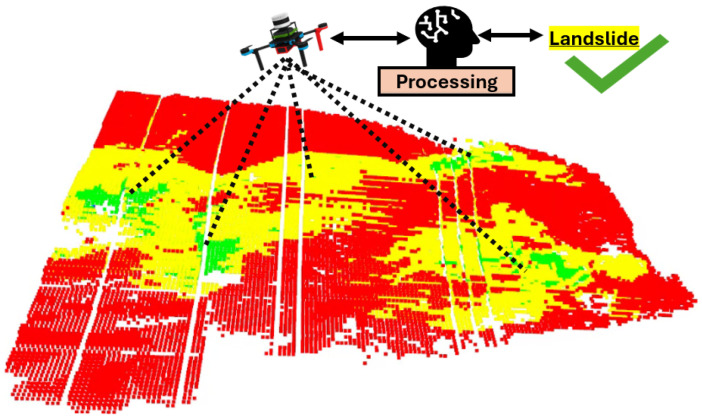
GeoFusion–3D instability classification for Cushetunk Mountain, NJ (Figure 8). At time t+1 (see Figure 2), the framework performs spatial clustering, geometric feature extraction, and anomaly-driven segmentation to identify unstable terrain regions. The resulting semantic map encodes stability levels as follows: red (stable terrain), yellow (weak instability), green (moderate instability), blue (high instability), and white (noise).

To evaluate robustness in stable terrain, we apply GeoFusion–3D to the undeformed Cushetunk Mountain dataset. As shown in Figure 11, the framework correctly classifies the majority of the terrain as stable (red), with minimal spurious detections.

This result highlights the effectiveness of the multi-scale fusion strategy (Equation (24)), which suppresses noise-driven anomalies while preserving true geomorphic structure.

Table 3 summarizes the hierarchy of geomorphic signatures consistently captured by GeoFusion–3D across all evaluated datasets. The framework successfully detects both fine-scale surface discontinuities and large-scale failure structures, demonstrating its ability to operate across multiple spatial resolutions.

These results highlight the effectiveness of combining point-level geometric anomalies (Equations (12) and (13)) with cluster-level contextual reasoning (Equation (20)). The resulting multi-scale representation enables robust separation between stable terrain and deformation-induced structures. Notably, the framework maintains stable performance under varying noise conditions, terrain resolutions, and geomorphic complexity, without requiring supervision or pre-event reference data.

## 6. Validity and Cross-Regional Evaluation

To assess cross-regional generalization and to situate geometric instability detection within a broader hazard-modeling framework, we evaluate GeoFusion–3D across four geologically diverse U.S. regions: **Wisconsin**, **North Dakota**, **South Dakota**, and **Arizona**. These terrains span glacial bluffs, sedimentary plateaus, depression-dominated mountainous regions, and arid desert slopes, thereby providing a stringent test of robustness beyond the controlled experimental datasets introduced earlier.

Because these cross-regional cases are not accompanied by field-confirmed landslide inventories, we do not treat the highlighted regions as definitive active landslides. Instead, they are interpreted as landslide-like geomorphic instability candidates. A detected region is described as landslide-like only when multiple geomorphic indicators co-occur: (i) scarp-like or arcuate boundary geometry, (ii) localized height drop or steep breakline, (iii) depressed or bowl-shaped source morphology, (iv) roughened or displaced downslope material, (v) curvature concentration or fracture-like discontinuity, and (vi) spatially coherent cluster-level support. Local discontinuities alone are therefore insufficient for a landslide interpretation, because similar point-cloud features may also arise from gully erosion, drainage incision, rock outcrops, road cuts, anthropogenic modification, vegetation-induced sampling gaps, or mesh/resampling artifacts. In the absence of field evidence, existing inventories, or multi-temporal confirmation, the role of GeoFusion–3D in these cases is to identify candidate instability zones that warrant expert geomorphic interpretation.

For broader contextual evaluation, we compare GeoFusion–3D with a recent physics-informed neural network (PINN) framework [33] designed for rainfall-induced landslide susceptibility modeling. The PINN estimates regional susceptibility by modeling subsurface soil-moisture dynamics through Richards’ equation and reports a macro F1 score of 0.88 across multiple U.S. locations. Importantly, the two frameworks address related but fundamentally different objectives. The PINN provides coarse-resolution, time-aggregated estimates of where landslides may develop based on environmental precursors, whereas GeoFusion–3D focuses on direct detection of observable geometric manifestations of failure from UAV LiDAR data at sub-meter resolution. The comparison is therefore intended to illustrate complementary operational characteristics.

Table 4 highlights the central conceptual distinction between the two approaches. The PINN captures *causal hydrological precursors* to instability, whereas GeoFusion–3D captures *observable geometric outcomes* of failure. These methods therefore operate at different stages of the landslide lifecycle and should be understood as complementary rather than mutually exclusive.

For this reason, the qualitative cross-regional analysis below should not be read as a replacement for field mapping or landslide inventory validation. Instead, it evaluates whether the same geometry-driven scoring logic produces plausible and spatially coherent candidate detections across different terrain types. The interpretation of each detected region is based on whether the high-confidence labels coincide with geomorphic patterns expected from slope failure, such as headscarp-like curvature, detachment boundaries, depressed source zones, displaced material, or runout-like roughened surfaces.

### 6.1. Arcadia, Wisconsin

**Figure 12:**
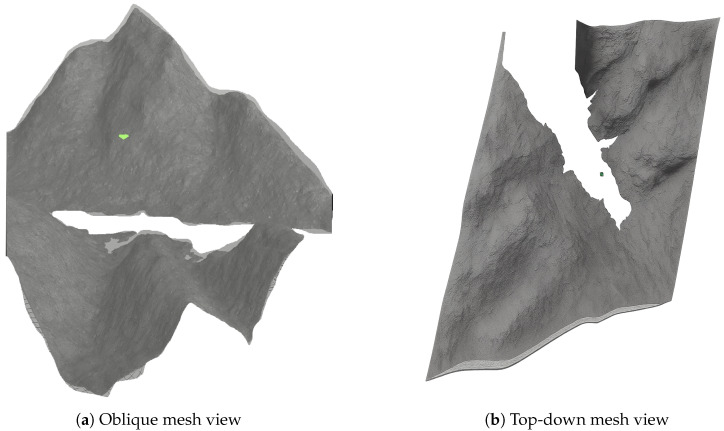
Arcadia, Wisconsin terrain reconstruction from UAV LiDAR.

**Figure 13:**
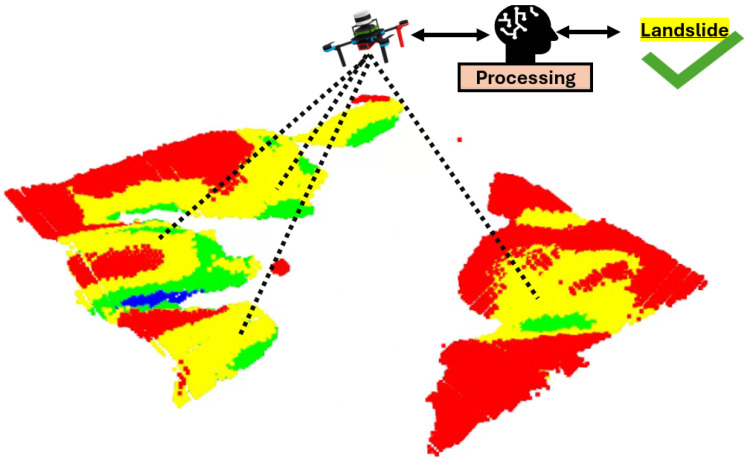
GeoFusion–3D instability classification for Arcadia, Wisconsin (Figure 13). At time t+1 (see Figure 2), the framework performs spatial clustering, geometric feature extraction, and anomaly-driven segmentation to identify unstable terrain regions. The resulting semantic map encodes stability levels as follows: red (stable terrain), yellow (weak instability), green (moderate instability), blue (high instability), and white (noise).

Figure 12 presents the Arcadia terrain from both oblique and top-down viewpoints, while Figure 13 shows the corresponding GeoFusion–3D instability field.

As shown in Figure 13, GeoFusion–3D highlights a continuous scarp boundary together with a central collapse basin, consistent with slope-failure morphology observed in Figure 12.

The Arcadia detection is interpreted as a landslide-like candidate because the high-confidence region is not an isolated rough patch. Instead, the detected area combines an arcuate scarp-like boundary, a central depressed basin, localized relief change, and spatially coherent cluster support. These criteria make the region geomorphically consistent with slope-failure morphology, although final confirmation would require field evidence, inventory comparison, or multi-temporal data.

In contrast, the PINN-based susceptibility predictions are dominated by low-risk labels when predicted at the coordinates for Arcadia terrain, indicating minimal estimated instability. This highlights the differing operational focus of hydrology-driven susceptibility models and geometry-driven detection frameworks. They are not designed to explicitly resolve localized geometric failure structures at sub-meter spatial resolution.

### 6.2. Sperati Point, North Dakota

**Figure 14:**
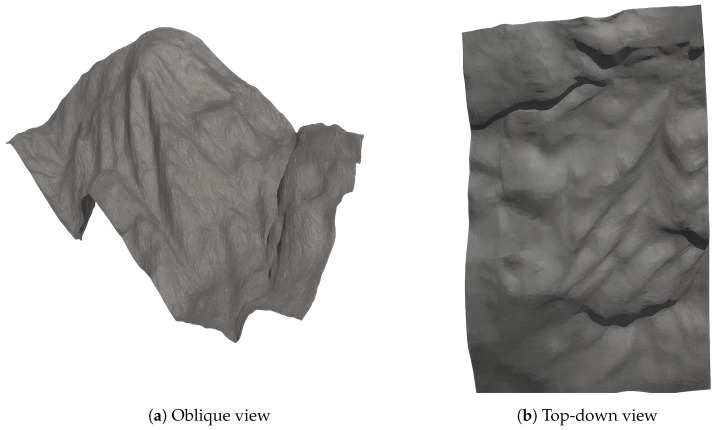
Sperati Point terrain, North Dakota. The terrain reconstruction is used for geomorphic interpretation only; detected discontinuities are interpreted as landslide-like candidates when they form coherent scarp, detachment, or displaced-material patterns rather than isolated roughness.

**Figure 15:**
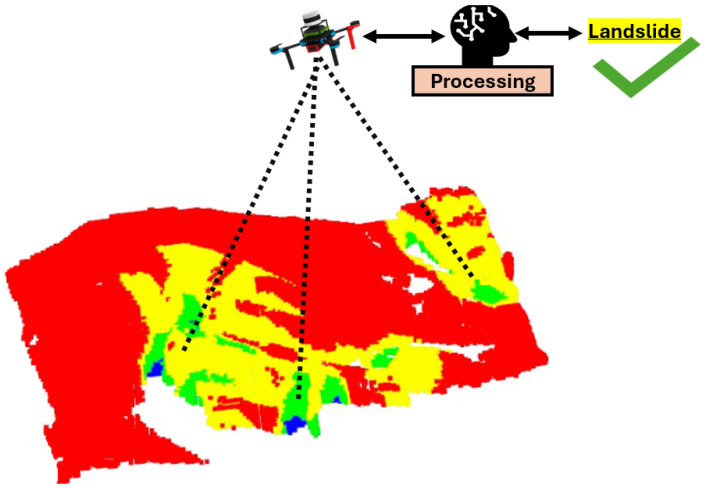
GeoFusion–3D instability classification for Sperati Point, North Dakota (Figure 15). At time t+1 (see Figure 2), the framework performs spatial clustering, geometric feature extraction, and anomaly-driven segmentation to identify unstable terrain regions. The resulting semantic map encodes stability levels as follows: red (stable terrain), yellow (weak instability), green (moderate instability), blue (high instability), and white (noise).

The Sperati Point terrain is shown in Figure 14, with the corresponding instability field in Figure 15.

Figure 15 reveals a semicircular high-instability structure that is morphologically consistent with bluff undercutting and block detachment. The detected region remains sharply localized and geometrically coherent, accurately delineating the failure boundary.

For Figure 14 and Figure 15, the interpretation as a landslide-like candidate is based on the joint presence of a semicircular scarp-like boundary, localized height discontinuity, and coherent high-confidence labeling along the apparent detachment margin. A simple drainage incision or gully may also produce a local depression, but it would typically appear as an elongated channel-like feature rather than a compact semicircular detachment zone with a sharply bounded unstable margin. Similarly, an isolated rock outcrop or point-cloud artifact may increase roughness locally, but it would not necessarily produce the same combination of curvature concentration, residual-depth anomaly, height drop, and cluster-level coherence. Therefore, GeoFusion–3D does not label the region as a confirmed landslide; it identifies the area as a candidate instability zone whose morphology is consistent with bluff undercutting or block detachment.

In contrast, the PINN-based susceptibility predictions are entirely dominated by low-risk labels when predicted at the coordinates for Sperati Point, indicating no predicted instability. This highlights the previously discussed key limitation of PINN-based approaches: while they model environmental susceptibility, they fail to capture localized geometric failure structures such as undercut scarps and detachment boundaries.

### 6.3. Thrall Mountain, South Dakota

**Figure 16:**
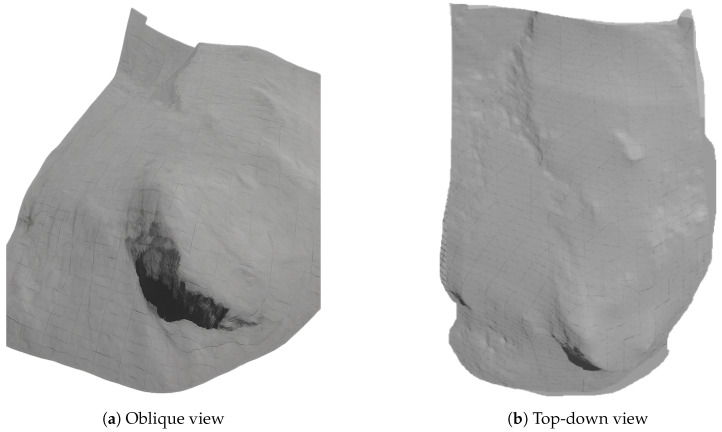
Thrall Mountain terrain reconstruction. The depression-dominated morphology is interpreted as a landslide-like candidate only where the detected region shows coherent basin geometry, localized height variation, and cluster-level instability support.

**Figure 17:**
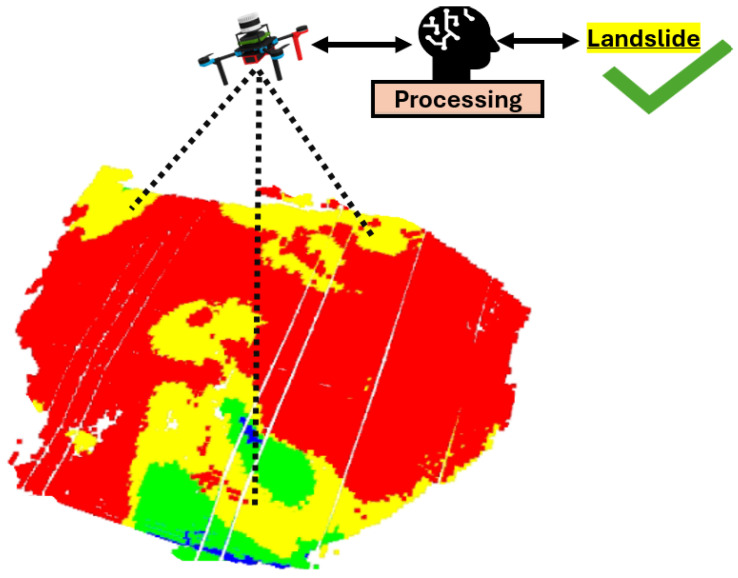
GeoFusion–3D instability classification for Thrall Mountain, South Dakota (Figure 17). At time t+1 (see Figure 2), the framework performs spatial clustering, geometric feature extraction, and anomaly-driven segmentation to identify unstable terrain regions. The resulting semantic map encodes stability levels as follows: red (stable terrain), yellow (weak instability), green (moderate instability), blue (high instability), and white (noise).

Thrall Mountain exhibits a broader, depression-dominated instability pattern, as shown in Figure 16 and Figure 17.

The instability field in Figure 17 shows a bowl-shaped high-confidence region surrounded by structured transition zones, consistent with retrogressive failure or localized material accumulation within a depression feature. GeoFusion–3D preserves the internal geometry of the disturbed region at high spatial resolution.

For Figure 16 and Figure 17, the detected area is interpreted using a stricter multi-feature criterion because depression-dominated terrain can also arise from ordinary erosional or drainage processes. The region is considered landslide-like only where the bowl-shaped depression co-occurs with localized height drop, curvature concentration around the basin margin, roughened or displaced internal material, and spatially coherent high-confidence labels. This helps distinguish a possible retrogressive failure or localized accumulation zone from a naturally concave valley floor, broad canyon morphology, or drainage incision. If only concavity or roughness were present without coherent residual-depth anomaly and cluster-level support, the region would be treated as ambiguous terrain roughness rather than a landslide-like candidate.

In contrast, the PINN-based susceptibility predictions are dominated by low-risk labels when predicted at the coordinates for Thrall Mountain, with only a small fraction indicating moderate susceptibility and no high-risk detections. This highlights a key limitation of hydrological frameworks: while they capture broader environmental susceptibility trends, they fail to resolve the internal geometric structure of failure regions.

### 6.4. Horse Shoe Hill, Arizona

**Figure 18:**
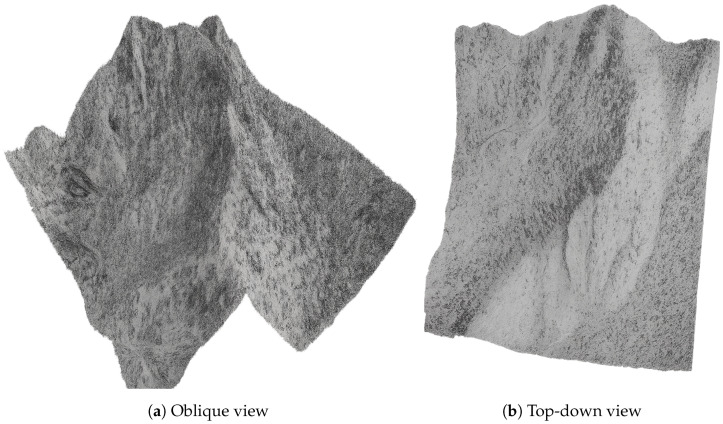
Horse Shoe Hill terrain reconstruction. Although the surface contains substantial natural roughness, roughness alone is not used as evidence of landslide activity; the corresponding instability field is interpreted through multi-feature agreement across residual depth, concavity, curvature concentration, height drop, and cluster coherence.

**Figure 19:**
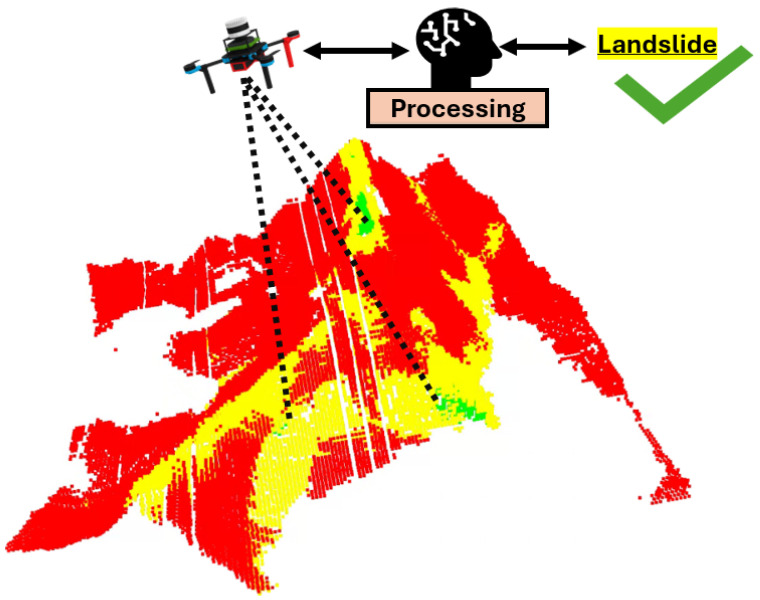
GeoFusion–3D instability classification for Horse Shoe Hill, Arizona (Figure 19). At time t+1 (see Figure 2), the framework performs spatial clustering, geometric feature extraction, and anomaly-driven segmentation to identify unstable terrain regions. The resulting semantic map encodes stability levels as follows: red (stable terrain), yellow (weak instability), green (moderate instability), blue (high instability), and white (noise).

Arizona provides a critical out-of-distribution validation case. The terrain and resulting instability field are shown in Figure 18 and Figure 19.

Unlike the wetter northern regions, Arizona exhibits weak hydrological susceptibility signals, and moisture-driven models naturally provide limited evidence of instability. However, Figure 19 shows that GeoFusion–3D identifies localized instability associated with surface fracturing and rock detachment.

Figure 18 contains substantial ordinary terrain roughness, which is why this case is important for clarifying roughness discrimination. GeoFusion–3D does not classify the entire rough surface as unstable. Instead, high-confidence labels are restricted to localized regions where roughness co-occurs with stronger geometric evidence: elevated local plane residuals indicating disrupted surface consistency, curvature concentration indicating fracture-like or breakline geometry, local height drop indicating relief discontinuity, and coherent cluster support indicating that the response is spatially organized rather than an isolated artifact. Ordinary rough desert terrain may produce high surface variability, but if it lacks concavity/depletion structure, sharp breakline geometry, and coherent cluster-level anomaly support, it remains red or weakly classified. Thus, the Arizona result should be interpreted as localized candidate rock detachment or fracture-related instability, not as a blanket labeling of all rough terrain as landslide-affected.

This interpretation also addresses possible non-landslide explanations. In arid terrain, high roughness may reflect rock outcrops, erosional remnants, drainage incision, or data artifacts. GeoFusion–3D reduces such false positives by requiring agreement between point-level signals and cluster-level geomorphometric context. Roughness contributes to the score only when it is accompanied by residual-depth anomaly, curvature concentration, height drop, and spatial coherence. Therefore, Figure 19 should be read as identifying candidate instability zones associated with localized surface fracturing and detachment-like morphology, while final attribution would require expert geomorphic interpretation or field validation.

In contrast, the PINN-based susceptibility predictions are dominated by low-risk labels, with no high-susceptibility detections. PINN-based models rely on hydrological signals and therefore fail to capture non-hydrological failure mechanisms prevalent in arid environments.

### 6.5. Cross-Regional Summary

Across all four regions, three consistent patterns emerge:

- In moisture-sensitive regions (WI, ND, SD), geometric detections broadly align with hydrological susceptibility trends.
- GeoFusion–3D preserves detailed failure morphology including scarps, depressions, and rupture boundaries at sub-meter resolution.
- In arid terrain (AZ), the method remains informative even when hydrological signals are weak or absent.

Across these cross-regional examples, the term “landslide” is therefore used in a candidate-detection sense unless field confirmation is available. The interpretation is strongest when detected regions exhibit a coherent set of geomorphic indicators, including scarp-like boundaries, localized height discontinuity, depressed source morphology, displaced or roughened downslope material, and cluster-scale spatial coherence. It is weaker when only one descriptor, such as roughness or concavity, is present. This distinction is important because local discontinuities in point clouds can arise from multiple geomorphic or non-geomorphic causes. GeoFusion–3D is designed to narrow the search space for likely instability features, while expert review, inventories, field evidence, or multi-temporal change detection remain necessary for definitive landslide classification.

Taken together, these results suggest that GeoFusion–3D can provide complementary geometric information alongside physics-based susceptibility models. While hydrological frameworks estimate environmental conditions associated with potential failure, the proposed method focuses on detecting observable terrain deformation and structural disruption directly from UAV-derived geometry. These capabilities may be particularly valuable in rapid post-event assessment scenarios where immediate geometric evidence is available but environmental histories or labeled inventories are limited.

### 6.6. Technical Comparison: PINN vs. UAV near Real-Time Detection

For clarity, we summarize the practical trade-offs below.

#### 6.6.1. Advantages of PINN

- Physically grounded modeling of soil-moisture evolution and infiltration processes.
- Incorporates environmental and climatic covariates unavailable to geometry-only methods.
- Strong reported performance for susceptibility classification (Macro F1 = 0.88).

#### 6.6.2. Limitations of PINN

- Cannot directly resolve geometric failure structures such as scarps, fracture boundaries, or detached blocks.
- Requires environmental data collection, supervised modeling, and offline processing.
- Operates at daily temporal resolution rather than near-real-time terrain sensing.

#### 6.6.3. Advantages of GeoFusion–3D

- Directly detects geometric failure signatures from raw LiDAR structure.
- Supports near-real-time, onboard-compatible inference.
- Operates independently of rainfall, soil, vegetation, or long-horizon environmental histories.

### 6.7. Comparison with State-of-the-Art 3D Models and Time Optimization

To further contextualize performance, we compare the proposed framework against representative 3D point-cloud learning baselines, including a graph neural network (GNN) [34], PointNet++ [35], and a multilayer perceptron (MLP) [36] regression baseline. Table 5 summarizes the results using $R^{2}$, RMSE, and mask IoU at a scar threshold of 0.5 blue per-point score $P_{i}$ (Equation (21)) and average them to understand the performance across all the test, and validation environments.

Despite being fully unsupervised, the proposed method achieves the strongest overall alignment with ground-truth scar structure, with an IoU of approximately 0.97. This result reflects the strength of the geometry-driven fusion strategy introduced in Section 3, where point-level anomaly signals are reinforced by cluster-level geomorphic context. In contrast, the supervised baselines require annotated training data and still produce lower spatial agreement, with the strongest alternative baseline (MLP) reaching an IoU of 0.9040. While the synthetic deformation experiments provide controlled evaluation with exact geometric ground truth, we acknowledge that such benchmarks do not fully replicate the complexity of natural landslide processes or constitute equivalent validation against independently mapped real-world inventories. The synthetic experiments are therefore intended primarily to assess geometric sensitivity, robustness to noise, and spatial delineation accuracy under controlled conditions, rather than to claim comprehensive real-world predictive validation.

From a computational standpoint, the complete pipeline executes in approximately 11.8 s for a 28 k-point cloud, including clustering, feature extraction, adaptive fusion, and scar delineation. This runtime supports near-real-time deployment during UAV surveys and demonstrates that high-quality landslide-scar extraction can be achieved without sacrificing operational feasibility.

### 6.8. Ablation Study and Parameter Sensitivity

To validate the design choices in Equations (19) and (20) and the fusion formulation in Equations (21)–(23), we conduct a systematic ablation study across three axes: (i) score calibration and interpretability thresholds, (ii) fusion weight constraints, and (iii) spatial context size in Phase 1 (Equation (1)).

Experiments are performed on both synthetic deformation datasets and real-world terrains described in Section 4, enabling controlled evaluation under known ground-truth conditions as well as generalization assessment across heterogeneous geomorphic regimes.

#### 6.8.1. Ablation on Confidence Thresholds

The qualitative interpretation of the continuous point-level score $P_{i}$ (Equation (21)) into discrete classes (red, yellow, green, blue) introduces thresholds at {0.50,0.60,0.70}. Although these thresholds are not used during inference, they are critical for interpretability and evaluation.

To justify these values, we analyze the empirical distribution of $P_{i}$ across all datasets. Because features are normalized via Equation (19), the resulting scores follow a bounded, approximately logistic distribution centered near 0.5. This is consistent with robust normalization practices for heavy-tailed geomorphic variables [37,38].

We perform percentile-based validation by comparing:

- $P_{i}<0.50$ (lower half of distribution),
- $0.50\leq P_{i}<0.60$ (mid-transition region),
- $0.60\leq P_{i}<0.70$ (upper-mid instability),
- $P_{i}\geq 0.70$ (top 20–25% of scores).

We observe that:

- Regions with $P_{i}\geq 0.70$ consistently align with injected scarps and displacement zones (Figure 9), indicating strong geomorphic coherence.
- The range $0.60\leq P_{i}<0.70$ captures transitional regions (e.g., deposition zones, shallow deformation).
- Values below 0.50 correspond to smooth, planar terrain with low depth anomaly (Equation (20)).

We further evaluate threshold sensitivity by sweeping decision cutoffs from 0.4 to 0.8 and computing overlap with synthetic ground truth. The IoU peaks in the range [0.65, 0.75], with minimal variation around 0.70, supporting the chosen categorization.

Thus, the thresholds are not arbitrary; they emerge from:

1. the logistic normalization in Equation (19),
2. empirical percentile separation of instability,
3. alignment with synthetic deformation ground truth.

#### 6.8.2. Ablation on Fusion Weight Constraint

The fusion formulation in Equation (20) combines cluster-level and point-level descriptors. The constraint

(28) $$w_{1}\geq 0.3$$

is introduced to ensure that cluster-level geomorphic context remains influential.

To evaluate this, we perform an ablation by varying $w_{1}\in \left[0.0,\;0.6\right]$ while optimizing remaining weights using the AUC-maximization procedure described in Section 3.4.

Results show:

- $w_{1}<0.2$: the model overfits to local signals ($d_{i}^{★}$, $\kappa_{i}^{★}$), resulting in noisy detections and increased false positives, especially in stable terrain (Figure 11).
- $w_{1}\approx 0.3$: optimal balance between global geomorphic structure and local anomaly detection.
- $w_{1}>0.5$: overly smooth predictions, missing fine-scale discontinuities.

This behavior reflects a known trade-off in spatial modeling: local features capture high-frequency variations, while aggregated descriptors encode structural coherence [16].

The value $w_{1}=0.3$ therefore represents the minimal threshold at which:

- cluster-level evidence remains dominant enough to suppress noise,
- point-level sensitivity is preserved for fine-scale detection.

Importantly, the constraint in Equation (23) is not a tuned constant but a lower-bound regularization ensuring stability across terrains.

#### 6.8.3. Ablation on ROI Size (Equation (1))

The region of interest (ROI) in Equation (1) defines a 100 m cube around the UAV. This parameter controls the trade-off between spatial context and computational efficiency.

We evaluate ROI sizes {50 m,75 m,100 m,150 m} and measure:

- detection accuracy (IoU),
- computational latency,
- cluster stability (DBSCAN consistency).

Findings:

- **50 m:** insufficient context; clusters fragment, reducing $P_{C}$ stability.
- **75 m:** improved performance but still limited for large scarps.
- **100 m:** optimal balance; stable clustering and full capture of geomorphic structures.
- **150 m:** marginal accuracy gain but significant computational overhead.

This aligns with LiDAR processing literature, where local spatial windows must exceed the characteristic scale of terrain features to preserve geomorphic continuity [17,18].

Thus, the 100 m ROI is selected as the smallest window that:

1. preserves cluster integrity,
2. captures full landslide morphology,
3. remains computationally feasible for onboard processing.

The ablation study confirms that the proposed design is not heuristic but grounded in:

- statistical normalization behavior (Equation (19)),
- multi-scale geomorphic representation (Equation (20)),
- spatial coherence constraints (Equation (1)),
- and empirical validation across synthetic and real terrains.

Notably, the interaction between cluster-level and point-level signals is essential. Removing either component leads to degraded performance, confirming the necessity of the multi-scale formulation illustrated.

### 6.9. Computational Efficiency and Inference Time Analysis

In our implementation, processing a typical UAV LiDAR scene consisting of approximately 5568 segments requires approximately 0.04 s using the full pipeline with segment matching enabled. The corresponding memory footprint is 0.186 MB.

These measurements demonstrate that the proposed method is computationally lightweight and capable of efficient inference under resource-constrained settings. In particular, the low latency and minimal memory requirements make the framework suitable for deployment on embedded or edge-computing hardware, including ARM-based systems with at least 4 cores, 1.8 GHz CPU frequency, and 2 GB RAM.

The measured runtime and memory footprint indicate that GeoFusion–3D is computationally lightweight for the tested point-cloud scenes, supporting rapid generation of instability maps in UAV-style processing workflows.

## 7. Discussion and Conclusions

This work introduced a fully unsupervised, geometry-driven framework for 3D landslide scar detection directly from UAV-mounted LiDAR data. By integrating voxel-level spatial organization, point-level geometric anomaly estimation, and cluster-level geomorphometric reasoning, the proposed method constructs a unified instability field capable of delineating landslide structures without labeled data, pre-event baselines, or rasterized terrain representations. It is important to emphasize that GeoFusion–3D addresses a fundamentally different problem formulation than traditional susceptibility modeling. Rather than estimating probabilistic conditions for future failure, the proposed framework focuses on directly extracting geometric evidence of terrain deformation from observed 3D structure.

Across controlled synthetic deformation benchmarks, an undeformed negative-control terrain, and terrain-derived cross-regional qualitative cases, the framework demonstrates consistent ability to highlight both large-scale failure-like structures and fine-scale geomorphic discontinuities. The combination of depth anomaly, concavity, and cluster-aware feature fusion enables robust separation between stable and unstable terrain, while the adaptive weighting mechanism emphasizes the most discriminative signals. Importantly, the stability-aware suppression strategy effectively reduces false positives in low-susceptibility regions, as validated on Cushetunk Mountain.

At the same time, GeoFusion–3D should be interpreted as a geometry-driven detector of landslide-like geomorphic instability candidates rather than as a definitive classifier of active landslides. The method identifies spatially coherent combinations of local plane residuals, concavity, curvature concentration, roughness, height drop, and cluster-scale disruption. These signatures are consistent with landslide scarps, depletion zones, displaced material, and collapse basins, but similar geometric patterns may also arise from non-landslide processes such as gully erosion, fluvial incision, rock outcrops, road cuts, anthropogenic excavation, vegetation-induced sampling gaps, or mesh/resampling artifacts. Therefore, high-confidence detections should be treated as candidate instability zones requiring geomorphic interpretation or independent validation when available. The value of the method lies in rapidly narrowing the search space for post-event inspection, rather than replacing field confirmation, expert mapping, or multi-temporal change detection.

Cross-regional evaluation further highlights the generalization capability of the approach. In moisture-sensitive regions, the detected geometric instabilities are broadly consistent with areas identified as susceptible in physics-informed models, reflecting shared environmental drivers of slope instability, while still capturing finer-scale structural deformation. This demonstrates that GeoFusion–3D can complement hydrology-based frameworks by identifying the observable manifestation of terrain failure at high spatial resolution and in near real time.

Despite operating in a fully unsupervised setting, the proposed framework achieves strong spatial agreement with ground-truth scar regions in synthetic benchmarks and maintains stable performance under varying terrain complexity, noise conditions, and deformation scales. These results suggest that the proposed framework is particularly suitable for automated landslide inventory generation and rapid post-event analysis in UAV-based workflows, where fast extraction of geometric deformation patterns is required.

We further acknowledge that a more comprehensive quantitative evaluation would benefit from direct comparisons with existing UAV LiDAR- or point-cloud-based landslide detection pipelines operating under similar sensing conditions. Such benchmarks would provide a more controlled assessment of geometric detection performance relative to related 3D methods. We identify this as an important direction for future work, particularly for standardized evaluation across point-cloud-based hazard detection frameworks. Future work will focus on extending the framework to multi-temporal LiDAR sequences for dynamic landslide evolution modeling and integrating temporal consistency constraints.integrating temporal consistency constraints. The main limitation is that the strongest quantitative evidence comes from controlled synthetic deformation with exact geometric ground truth; therefore, future work should validate GeoFusion–3D against independent UAV-LiDAR landslide inventories and multi-temporal field surveys.

## Figures and Tables

**Table 1:** Experimental sites, data provenance, and validation objectives.

Site	Terrain	Data Provenance	Validation Role	Reason for Selection
Washington Creek	Alpine, high-relief	Mesh-derived terrain with controlled Blender deformation and LiDAR-like resampling	Recovery of known scar-like deformation	Tests detection in steep, complex terrain with pronounced scarps and sharp breaklines
Kincaid Park	Coastal bluff	Mesh-derived terrain with controlled shallow deformation and LiDAR-like resampling	Detection of subtle, low-amplitude deformation	Tests sensitivity to weak concavity and diffuse bluff-failure morphology
Cushetunk Mountain	Basalt ridge	Unmodified terrain-derived point cloud	Negative-control/false-positive suppression	Tests whether rough but stable terrain is incorrectly labeled as unstable
Cross-regional qualitative cases	Mixed regional terrains	Terrain-derived point clouds processed with the same simulation-resampled pipeline	Qualitative transferability analysis	Tests whether the same geometry-driven scoring highlights plausible instability candidates across additional terrain types

**Table 2:** Summary of GeoFusion–3D evaluation outcomes across validation regimes.

Case	Validation Type	Main Evidence	Observed Outcome
Washington Creek	Controlled synthetic deformation	Known injected scar-like target; visual alignment; IoU-based evaluation	High-confidence regions align with injected scarp-like deformation and displaced material.
Kincaid Park	Controlled shallow synthetic deformation	Known low-amplitude deformation; visual alignment	Detects diffuse bluff-failure-like deformation through concavity, residual depth, and cluster coherence.
Cushetunk Mountain	Negative-control terrain	No injected deformation	Majority of terrain remains stable, supporting false-positive suppression in rough but stable terrain.
Arcadia, WI	Cross-regional qualitative case	Candidate geomorphic interpretation	Highlights coherent scarp-like boundary and collapse-basin morphology.
Sperati Point, ND	Cross-regional qualitative case	Candidate geomorphic interpretation	Highlights bluff-like arcuate instability candidate with coherent cluster support.
Thrall Mountain, SD	Cross-regional qualitative case	Candidate geomorphic interpretation	Highlights bowl-shaped/depression-like candidate region.
Horse Shoe Hill, AZ	Cross-regional qualitative case	Candidate geomorphic interpretation	Restricts high-confidence detections to localized rough/fracture-like regions rather than labeling all rough terrain.

**Table 3:** Observed geomorphic signatures captured by GeoFusion–3D across all datasets.

Feature Type	Observed Behavior
Fine-scale discontinuities	Detected along shear edges, fracture lines, and localized breaklines at cm–dm scale resolution.
Large headscarps	Clearly delineated in high-relief terrain with sharp spatial boundaries and strong cluster-level confidence.
Translational block movement	Identified through coherent separation patterns and downslope displacement structures.
Deposition zones and toe bulging	Captured in synthetic deformation scenarios, including runout accumulation and material buildup regions.

**Table 4:** Conceptual comparison between the PINN susceptibility framework and GeoFusion–3D, highlighting complementary operational objectives and sensing modalities.

Aspect	PINN [33]	GeoFusion–3D
Objective	Landslide susceptibility prediction	Direct landslide-scar detection
Input	Soil, weather, vegetation, and spatial covariates	Raw UAV LiDAR geometry
Output	Regional susceptibility map	Point-level instability field and scar delineation
Resolution	Coarse, grid-based	Sub-meter, point-cloud based
Operation	Offline, daily aggregation	Near-real-time onboard-compatible
Reported performance	Macro F1 = 0.88	IoU ≈0.97 on synthetic scars
Primary strength	Hydrological precursor modeling	Structural failure detection
Primary limitation	Cannot directly resolve scar geometry	Does not explicitly model rainfall physics

**Table 5:** Performance comparison of baseline 3D point-cloud models and the proposed method for blue per point score.

Model	$R^{2}$	RMSE	IoU (Scar ≥0.5)
GNN	0.8743	0.032651	0.8889
PointNet++	0.8256	0.038450	0.8660
MLP	0.8962	0.029666	0.9040
GeoFusion-3D	0.9789	≈0.002	≈0.97

## Data Availability

The original contributions presented in this study are included in the article. Further inquiries can be directed to the corresponding author.

## Abbreviations

The following abbreviations are used in this manuscript:

UAV	Unmanned Aerial Vehicle
LiDAR	Light Detection and Ranging
ROI	Region of Interest
DBSCAN	Density-Based Spatial Clustering of Applications with Noise
PCA	Principal Component Analysis
IQR	Interquartile Range
IoU	Intersection over Union
PINN	Physics-Informed Neural Network
DEM	Digital Elevation Model
TLS	Terrestrial Laser Scanning
ML	Machine Learning
LSE	Latent Spatial Effect
3D	Three-Dimensional
2D	Two-Dimensional
